# Evaluating urban environmental and ecological landscape characteristics as a function of land-sharing-sparing, urbanity and scale

**DOI:** 10.1371/journal.pone.0215796

**Published:** 2019-07-25

**Authors:** Matthew Dennis, Katherine L. Scaletta, Philip James

**Affiliations:** 1 Department of Geography, School of Environment Education and Development, University of Manchester, Manchester, United Kingdom; 2 School of Science, Engineering and Environment, University of Salford, The Crescent, Salford, United Kingdom; Curtin University, AUSTRALIA

## Abstract

Within urban landscape planning, debate continues around the relative merits of land-sharing (sprawl) and land-sparing (compaction) scenarios. Using three of the ten districts in Greater Manchester (UK) as a case-study, we present a landscape approach to mapping green infrastructure and variation in social-ecological-environmental conditions as a function of land sharing and sparing. We do so for the landscape as a whole and in a more focussed approach for areas of high and low urbanity. Results imply potential trade-offs between land-sharing-sparing scenarios relevant to characteristics critical to urban resilience such as landscape connectivity and diversity, air quality, surface temperature, and access to green space. These trade-offs are complex due to the parallel influence of patch attributes such as land-cover and size and imply that both ecological restoration and spatial planning have a role to play in reconciling tensions between land-sharing and sparing strategies.

## Introduction

### Green infrastructure, urbanisation and land-sharing-land-sparing

The concept of green infrastructure has emerged as a promising framework to understand, manage and enhance the multiple benefits delivered from nature, particularly in highly fragmented landscapes [1]. A green infrastructure approach involves optimizing multi-functionality in terms of social, ecological and economic benefits [2] and seeking resilience through landscape diversity, connectivity and micro-climate regulation [3]. With the unabated growth of urban areas in terms of population and the associated sprawl (land-sharing) of developed areas into the rural hinterland, debates surrounding the optimum spatial configuration on which to base urban planning persist. At the centre of this debate is a tension between the relative social-ecological effects of urban densification (or the so-called compact cities approach–land sparing) versus urban sprawl. Resolving such tensions and illuminating pathways towards sustainable cities, which support human well-being, ecological diversity and climate action, addresses multiple Sustainable Development Goals and is at the heart of achieving Goal 11: Sustainable Cities [4].

In recent years, a model, borrowed from landscape ecological studies on the effects of agricultural land-use on biodiversity [5], has been adopted as a means to explore the influence of urbanization on ecological integrity. This land-sharing (sprawl) versus land-sparing (compaction) model is particularly useful in the context of urbanization given the parallels that exist between the latter and agriculture-driven land-use change on which the concept was originally founded, namely high levels of local species extinction and ecosystem service degradation [6]. In an urban context, land-sharing implies the promotion of lower-density development which leads to smaller, more fragmented patches of public green-space and greater cover by private domestic gardens. Conversely, a land-sparing approach is promoted in cases where non-green land-use is compacted in order to allow for larger patches of green-space. This template theoretically favours large public green spaces ahead of smaller private green spaces in the form of domestic gardens [7]. However, this dichotomy of public and private green land-use is still poorly understood from ecological, social and environmental points of view. Moreover, there is, as yet, insufficient evidence that public or private green land-use *per se* promotes either sharing or sparing outcomes. This situation is the result of previous studies on urban land-sharing-sparing outcomes, and on urban land-use change more generally, focussing on simplified land-use metrics. For example, urban sharing-sparing studies have typically adopted housing density as a proxy for urbanization intensity [8], [9]. This suggests an implicit bias towards the effect of private land-use and associated green-space configurations whereas the broader field of research into urban growth and land-use dynamics generally proposes large-scale processes or socio-economic factors as key drivers of change [10], [11].

Another important but under-considered dichotomy inherent in human-dominated landscapes involves the distinction between land-use and land-cover. A key shortcoming of both the conceptualization and spatial representation of green infrastructure in research on urban areas is a singular consideration of green-space as either land-use (i.e. its function) or land-cover (i.e. its physical-ecological form). In addition, many authors use these terms interchangeably [2], [12],[13], or subsume characteristics of both under a single measure, thereby losing important information (e.g. [10], [14]). In order to understand the relative benefits of land-sharing versus sparing in urban areas, and the influence of individual land-uses and their associated land-cover, integrated datasets are required. For example, the degree to which urban areas reflect land-sharing or land-sparing configurations is necessarily the result of land-cover, whereas planning strategies generally reflect decisions on land-use. Recent work has highlighted the potential hazards of conflating green land-use with land-cover in urban planning [15] where vegetation cover may be significantly over-estimated for certain land-uses. Moreover, with more integrated datasets, the assumptions around the role of public versus private urban green-space in promoting sharing and sparing scenarios respectively can also be clarified, which should inform persisting debates within urban planning.

### The urban-to-peri-urban context

The spatial and temporal heterogeneity of landscapes subject to urbanisation stand in contrast to the relatively homogenizing effect of land-use change by agriculture and reinforce the need for high resolution, integrated data on urban spatial configurations. Gradients of urbanisation in particular create complex social-ecological conditions. Rural to urban gradients have been shown to exhibit considerable variation in ecosystem service provision [16], [17], well-being effects of green-space [18] and biodiversity outcomes [19]. Moreover, urbanised landscapes covering city-regions may encompass a range of human-dominated land-uses from highly compacted urban centres to low-density suburbs as well as agricultural landscapes in the peri-urban fringe. Due to such contrasting land-use-land-cover configurations, calls have rightly been made to employ whole-landscape approaches to modelling sharing-sparing outcomes in urban systems [6]. In addition to whole-landscape assessments we also argue that analyses at sub-landscape scales, for example within urban and peri-urban zones, are necessary given that the subject of a land-sharing-sparing model (i.e. the land being “spared”) will differ depending on the context. For example, taking a sparing approach in high-urban areas will typically imply the promotion of urban intensification towards consolidating larger patches of urban green-space whereas, in peri-urban areas, the “spared” land will likely take the form of agricultural or forestry land. This raises another important point related to the land-sharing-sparing model within the context of urbanisation. Much of the debate and associated research related to land-sharing and sparing in agricultural landscapes is predicated on the relative success of modelled yield-species density curves within biodiversity supporting habitats. However, many peri-urban landscapes typically comprise already degraded ecosystems in various stages of agricultural land-use. Indeed, for some functional groups, urban areas, and residential gardens in particular, can contain higher diversity and abundance than the agricultural hinterland [20]. Therefore, it is entirely possible that assumptions applied to land-sparing conservation efforts in areas containing intact biodiversity-supporting vegetation, may not be applicable to landscapes made up of complex juxtapositions of highly-modified land-uses. Given the variance in green infrastructure function, heterogeneity and quality between urban and peri-urban areas, information on vegetation type and health is a critical factor (along with spatial characteristics such as connectivity and patch size) when judging the productivity [21] and resilience (*sensu* Ahern, [22]) of landscapes characterised by (semi-)natural and highly modified habitats. We suggest that these simple but highly relevant dichotomies (public and private; land-use and land-cover; high and low urbanity) may provide useful points of departure in order to explore complexities inherent in the broader conceptual separation of land-sparing and land-sparing approaches.

Despite the need for integrated conceptualisations of urban landscapes, research on urban land sharing and sparing has largely sought to reduce the complex characteristics of urban areas. For example, studies have typically modelled hypothetical landscapes based on observed patterns of species distribution [8] as a response to broad land-use types such as building density [23]. In addition, meta-analyses drawing on a range of geographically diverse studies [24] have been carried out in order to identify common trends. These reductionist approaches however, have not considered wider social-ecological factors such as landscape connectivity, heterogeneity, overall green cover quantity and quality or other socio-environmental factors such as access to nature, urban cooling or air quality. We argue that a more holistic approach to evaluating urban landscapes is necessary in order to inform planning frameworks that align with UN Sustainable Development Goals. The creation of landscapes that promote human well-being and urban resilience to climate change, and which address inequalities in addition to biodiversity loss, requires a green infrastructure approach which considers a range of social-ecological outcomes [3], [25], [26], [27]. Such an approach is particularly pertinent in developing nations within which the highest levels of conversion to urban land-use, as well as high levels of environmental inequality, can be found [10], [28], [29]. However, most studies from which knowledge on urban land- sharing-sparing outcomes are based have been carried out in the global north within developed countries. Furthermore, despite the threat of climate change [30], rises in chronic health conditions [31], [32], [33] and the extinction of experience of nature [34] in the urban global north, few studies have paid attention to these broader environmental considerations in their assessments. More research is therefore necessary in order to understand the system-wide implications of urban development along the land-sharing-sparing spectrum. For example, it is true that increased urban development is broadly linked to greater human well-being [35]. Studies based on post-industrial cities may, however, provide insights from which recently developing nations, with more relaxed or non-existent urban planning regulations, may learn in order to better navigate the social and ecological challenges implied by the urbanisation process.

A land-sharing-land-sparing dichotomy (and associated notions of sprawl/compaction) and knowledge of related outcomes may provide an accessible template for ecologists and planning authorities to engage with sustainable urbanisation. For example, a range of metrics have been put forward in efforts to understand how urban green spaces and associated urban form may influence resilience to increased urban heating [14]. The multiplicity of such indices and the difficulty in interpretation which many of them imply has been recognised by other authors who have sought to simplify such knowledge generation through the use of data reduction techniques [36]. Such means necessarily result in a loss of information and composite indices are, by definition, less easily translated into practice. In contrast, we argue that the land-sharing-sparing paradigm represents a potentially accessible means to both conceptualise and operationalise urban landscape planning. Moreover, the simplicity of the model means that it can be as readily applied to environmental characteristics such as urban heating, air quality and access to green-space as it has been to biodiversity metrics.

### Translating land-sharing-sparing outcomes into practice-oriented outputs

The consideration of wider characteristics such as overall green cover and quality in urban localities is particularly important if urban studies are to be based on the same robust logic as agriculture-based studies on land-sharing -sparing. The latter are assessed primarily at the level of yield-to-species density performance in order to compare the relative success of sharing-to-sparing scenarios [37]. In urban areas however, the management goal is less clear or, at least, characterised with less consistency. Although housing density provides a useful proxy for level of development in urban environments [8], [34], this comprises only one type of built infrastructure common in urbanizing landscapes. Sophisticated measures of “yield” from urbanisation, comparable to the use of the term in agricultural land-sharing-sparing models, are not forthcoming. This is likely in part due to the multiplicity of societal gains afforded by urbanisation (e.g. knowledge, skills, employment, innovation: [38], [39]), relative to agriculture land. Therefore, we argue that in the urban context, where measuring productivity is a more complex issue, in order to assess the relative performance of land that remains undeveloped, a logical approach is to standardise comparisons of land-sharing and land-sparing scenarios by the degree of development and scale. The former requires that, for the same degree of urban development (e.g. surface sealing) a direct comparison across a range of desirable landscape attributes can be made between different spatial configurations. This is important for three reasons. Firstly, without this standardised approach, it is not possible to assess whether relative gains (e.g. land-cover diversity and connectivity) are due to spatial factors or simply a greater amount of green land-cover. Secondly, by taking a standardised approach, meaningful comparisons across scales of investigation are thereby permitted. By developing assessments which model outcomes across scales and are standardised by area, a more informed view can be taken on spatial planning approaches which balance land-use productivity with landscape resilience. Thirdly, decision-makers are required to develop urban spatial frameworks within defined spatial extents according to administrative boundaries. Therefore, research which can identify optimum landscape configurations for a given degree of development at a range of scales are desperately needed in order to allow planners to design urban areas which can provide essential ecosystem services to local residents. Such knowledge may assist decision-makers to identify bottom lines for the amount of green infrastructure cover necessary at a range of scales that, when consisting of suitable type and distribution, promote productive, sustainable landscapes.

To our knowledge, no studies on land-sharing-sparing scenarios exist that extensively and accurately characterise urban green infrastructure of whole landscapes. The latter is important in order to model ecological and socio-environmental factors vital to sustainable urban planning. For example, from an ecological perspective, landscape connectivity and heterogeneity are positively linked to both the provision [21] and, in particular, the resilience [22] of ecosystem services, whereas attributes such as core area and primary productivity are likewise important indicators of ecosystem service providing landscapes [40], [41]. From the perspective of urban residents, access to green spaces [42], urban cooling [40] and air quality [43] are all important quality of life factors which may be moderated by the configuration of urban landscapes. In order to create suitable data capable of achieving an integrated assessment of land-sharing-sparing outcomes for a range of urban-relevant processes, a novel spatial dataset was created, following a method developed by [44]. This method allowed the precise measurement of land-use-land-cover combinations across a spatially contiguous urban area comprising the two cities of Manchester and Salford, and the Metropolitan Borough of Trafford, all parts of Greater Manchester, in north-west England, UK. Using this integrated dataset, our overall aim was to evaluate associations between sharing-sparing scenarios on a range of social-ecological-environmental factors relevant to urban landscape productivity and resilience. In order to do this robustly we focussed on potential mediating factors and, as such, our objectives were three-fold: 1: to assess the relative contribution of land-use-land-cover combinations to sharing-sparing configurations; 2: to evaluate the relevance of urban and peri-urban contexts in assessing the relative merits of different landscape configurations, and 3: to identify scale-effects in the performance of sharing-sparing scenarios.

## Methods

### Spatial data on land-use and land-cover

A composite spatial dataset covering the contiguous urban areas of three districts in Greater Manchester (the cities of Manchester, Salford and the metropolitan borough of Trafford) was achieved through a combination of remote sensing and GIS techniques based on a method published by Dennis et al. [44]. Briefly, the method achieves the characterisation of discrete landscape features through an integration of land-use and land-cover data. Land-use (from OS Mastermap Topography and Greenspace layers 2017, [45], [46] and UK Land Cover Map 2015, [47]) was computed for public (including all public parks and amenity green spaces), domestic green-space (private gardens including rented allotment gardens), urban fabric, informal urban greenery (street-scapes and informal and/or spontaneous vegetation within the urban fabric), institutional land and peri-urban land-use within the study area. In addition, spatially co-incident data on land-cover were classified through Planet Scope 3 m imagery, 2017 [48] and supplemented by Ordnance Survey Rivers, Woodland and Buildings layers (OS Open Rivers 2018, [49] OS Open Map Local 2018, [50]) and City of Trees Tree Audit 2011 data, [51], resulting in five classes (built, ground vegetation, field layer vegetation, tree canopy and water). Table 1 contains metadata information for spatial data layers used in this study.

**Table 1:** Spatial datasets used in this study.

Name	Use in this study	Source/Year	Data model format	Resolution(raster)/minimum mapping units(vector)
Topography Layer	Extraction of garden polygons	Ordnance Survey 2017	Vector	1 m^2^
Green-space Layer	Extraction of green-space land-use polygons	Ordnance Survey 2017	Vector	1 m^2^
UK Land Cover Map	Demarcation of urban and peri-urban areas	Centre for Ecology and Hydrology 2015	Vector	0.5 ha
Open Map Local	Extraction of woodland and buildings polygons	Ordnance Survey 2019	Vector	1 m^2^
OS Open Rivers	Extraction of rivers and lakes	Ordnance Survey 2019	Vector	1 m^2^
Greater Manchester Tree Audit data	Treeline and canopy cover polygons	City of Trees 2011	Vector	1 m^2^
PlanetScope 3 m 4-band satellite imagery	Supervised classification of ground and shrub vegetation and built surfaces; calculation f NDVI	Planet.com, 2017	Raster	3 m
PopGrid	Population data as number of residents 100 m^-2^	University of Southampton	Raster	10 m

**Fig 1:**
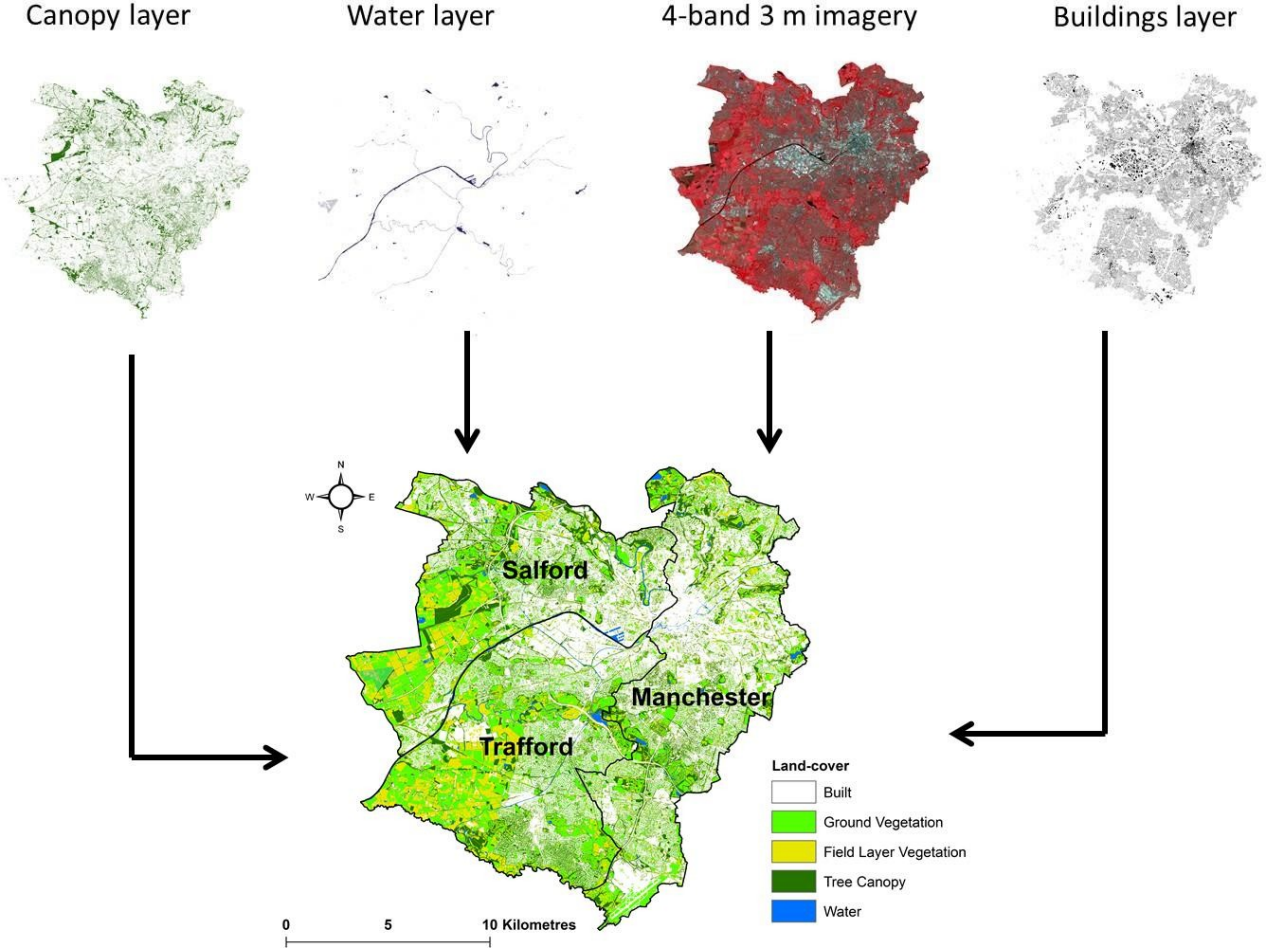
Work-flow for the land-cover classification used in this study combining 3 m satellite imagery (Planet, 2017), tree canopy data (City of Trees 2011 and Ordnance Survey Open Map Local, 2018) and buildings data (OS Open Map Local, 2018). Contains OS data Crown copyright and database right 2019 Ordnance Survey (100025252).

**Fig 2:**
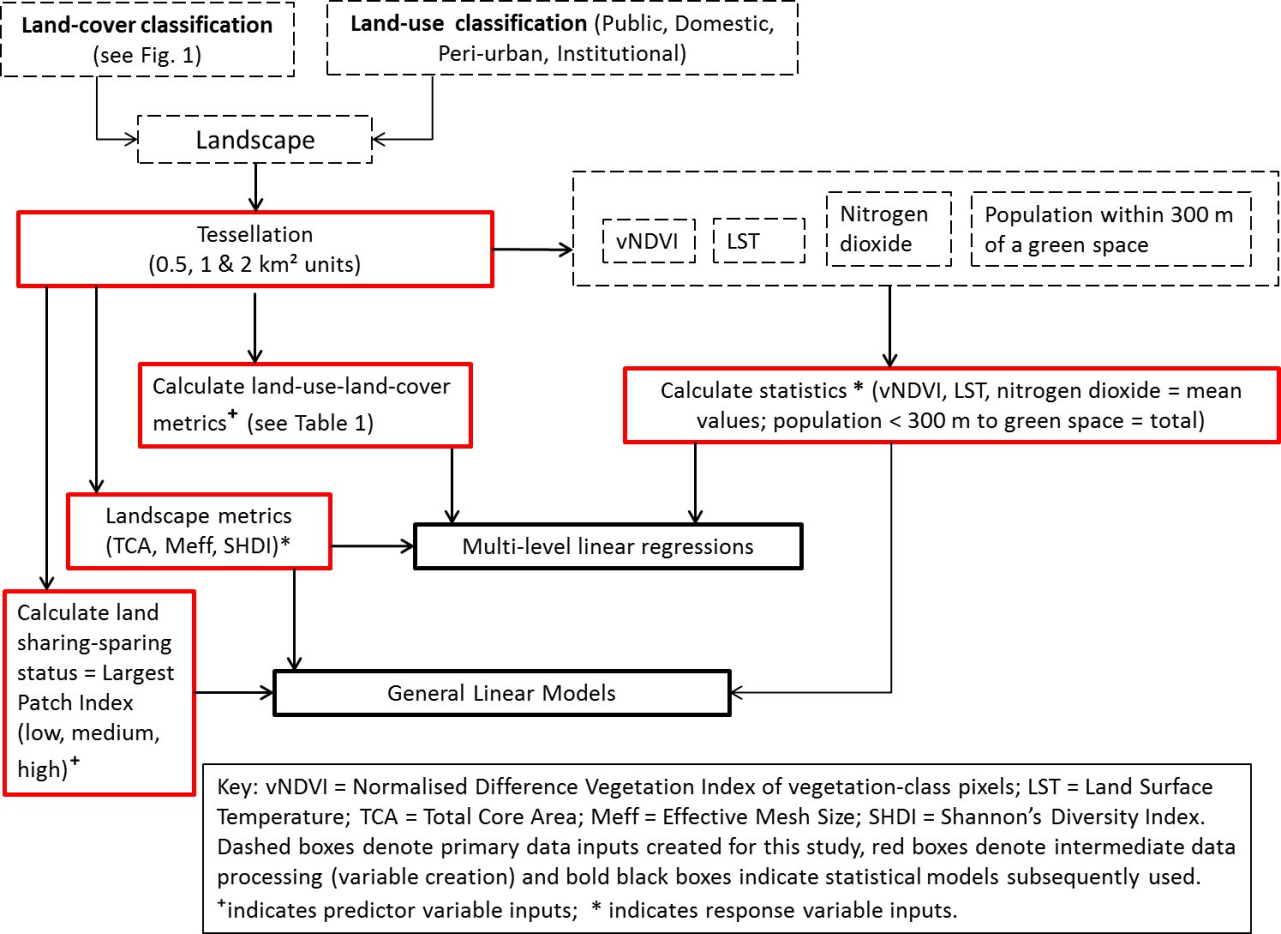
Workflow of analytical steps carried out within this study.

Accuracy assessment of the land-cover layer was achieved through 200 randomly generated sampling points (40 for each land cover type) for which classified values were cross-tabulated with ground checking evaluations using Edina 2017 aerial photography [52]. Overall accuracy and Cohen’s Kappa co-efficient were subsequently calculated. The work flow for the land-cover classification is summarised in Fig 1. A visual summary of all steps in the analysis carried out is provided in Fig 2.

### Landscape and environmental metrics

**Fig 3:**
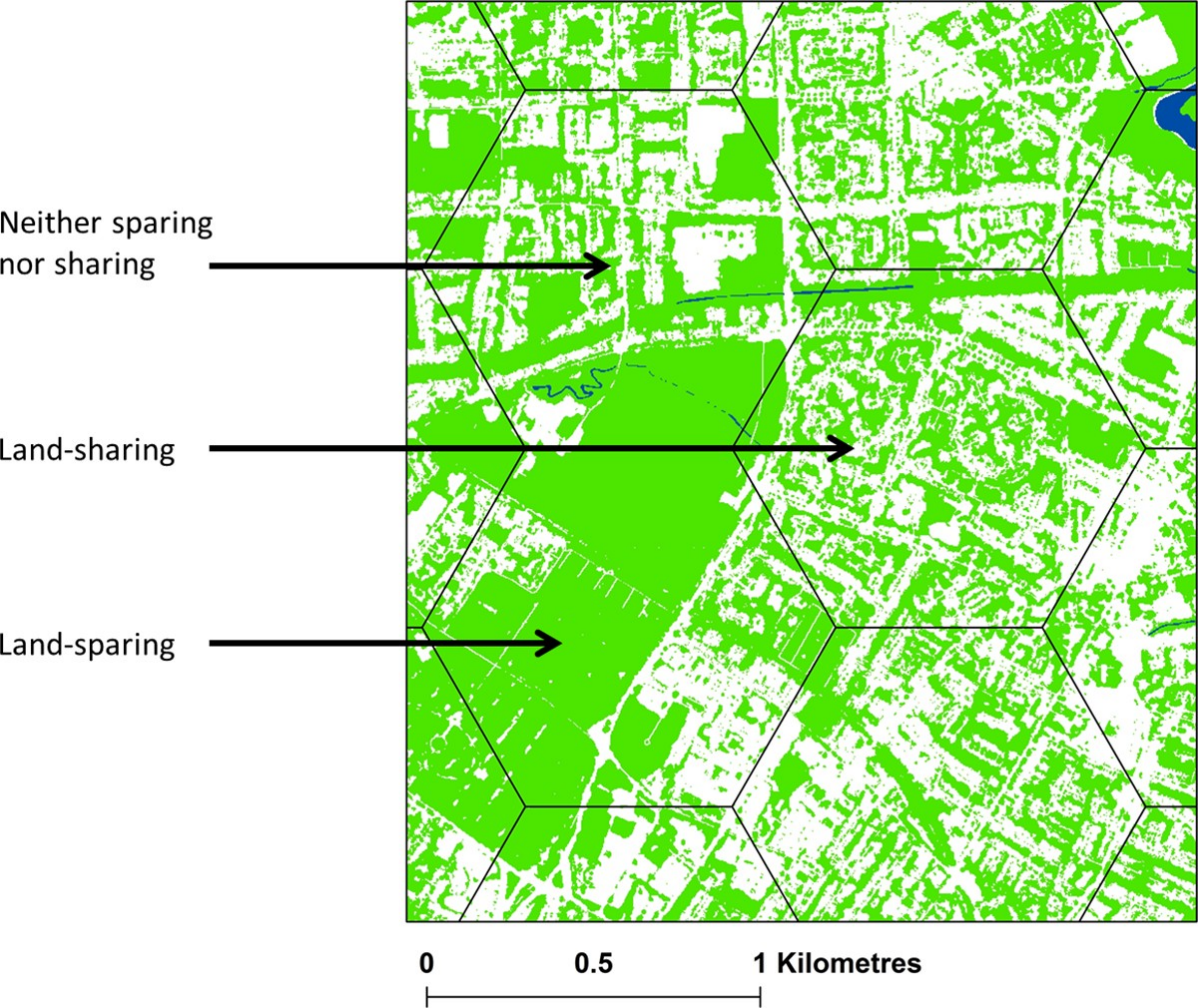
Example of areas classified as land-sharing, land-sparing and neither sharing nor sparing (contains City of Trees, 2011 data, Planet, 2017 data and OS Open Rivers data, 2018). Contains OS data Crown copyright and database right 2019 Ordnance Survey (100025252).

A range of social-ecological metrics were quantified within 0.5, 1 and 2 km^2^ zones created through a hexagonal tessellation of the study area using the Sampling toolset within ArcMap 10.4.1 (this shape was used as it maximised cover of the study area and provided the greatest number of cases for analysis). We chose 1 km^2^ as a commonly adopted unit of analysis in ecological studies such as species distribution and diversity modelling [53], [54]. Repeated analyses at 0.5 and 2 km^2^ thereby allowed us to explore both scale-effects and the efficacy of such units for evaluating other socio-environmental outcomes. The land-cover layer was used to compute a range of landscape characteristics including effective mesh size (Meff), total core area (TCA), largest patch index (LPI) and Shannon’s land-cover diversity (SHDI), calculated using the QGIS plug-in Lecos [55]. Values for Meff and TCA are returned in the spatial units of the source data and, in order to allow comparability across scales, these were standardized as a percentage of the spatial units used in our analysis. In addition, socio-environmental variables land surface temperature (LST), background nitrogen dioxide concentration and population within 300 m of a recreational green-space were calculated. LST was derived from Landsat 8 TIRS imagery (July 2018 at 30 m resolution, [56]) based on methods outlined by Sobrino et al. [57] and Advan and Jovanovska [58] see S1 Appendix for details), background nitrogen dioxide concentrations were interpolated using the ordinary kriging method from Defra 2018 background nitrogen dioxide data points, [59] and population within 300 m of a recreational green-space was calculated by summing population counts (using PopGrid 10 m population data [60]) within buffers applied to green-space boundaries. As a measure of vegetation quality, the normalized difference vegetation index (NDVI) was calculated for pixels in the dataset classified as vegetation (i.e. ground layer, field layer and tree canopy). This was achieved by creating a mask based on all green land-cover pixels and setting this as the environment for the NDVI calculation within ArcMap (version 10.4), again at units of 0.5, 1 and 2 km^2^. We refer to this metric as vNDVI in this paper. Subsequently, the degree to which the tessellated regions exhibited land-cover indicative of land-sharing or land-sparing was judged according to their largest patch index (LPI), following similar approaches taken elsewhere [34]. This metric represents the proportion of green-space in a given locality that is comprised of a single contiguous patch. High values, therefore, represent increasingly large (i.e. spared) patches relative to overall cover by green-space. Tessellated regions were divided into three quantile groups representing low (land-sharing), medium (neither land-sharing nor land-sparing) and high (land-sparing) scores for LPI. Fig 3 gives examples of areas exhibiting low, medium and high LPI (land-sharing, neither sharing nor sparing, and land-sparing respectively).

The influence of land-sharing-sparing on critical ecological and socio-environmental attributes was assessed through a series of general linear models using the three LPI quantile groups as fixed factors. Meff, SHDI, TCA, vNDVI, LST, nitrogen dioxide and total population within 300 m of a recreational green-space were all entered as dependent variables whilst controlling for total green land-cover. Controlling for overall green cover was equally important from a practical as well as a methodological point of view. LPI and total green land-cover were significantly correlated (at units of 1 km^2^, for example, Pearson’s r = 0.82; p < 0.01). Therefore, entering green land-cover as a co-variate ensured that the LPI metric was not acting as a surrogate for the former in our assessments. Analyses were repeated at low and high urbanity levels (separated by the median values of developed land–i.e. non-green land-use—within each of the 0.5, 1 and 2 km^2^ units of analysis).

Given that socio-economic status is known to influence green cover in urban land-uses [15], [44] and that the latter may influence the performance of sharing-sparing patterns of green infrastructure, information on vegetation cover within green land-uses was calculated for different economic groups. Income deprivation scores from the English Indices of Multiple Deprivation [61] were downloaded for Lower Super Output Areas (LSOAs; English census reporting units–mean population is 1500) and mean values were calculated for the smallest unit of analysis for this study (0.5 km^2^ zones; N = 554) in order to best reflect the spatial variance in the LSOA boundary data (N = 570; mean area = 0.56 km^2^). This provided a socio-economic context within which to further evaluate our results. Finally, associations between land-use-land-cover metrics and social-ecological outcomes were explored through multiple linear regression analysis. LPI, TCA, Meff, SHDI, mean LST, mean nitrogen dioxide and mean vNDVI values were entered as dependent variables. The list of land-use-land-cover metrics computed and entered into regression models as independent variables is given in Table 2.

**Table 2:** Descriptions of landscape metrics computed for use in linear regression analyses within this study.

Name	Description	Expressed as:
Domestic	Domestic green space	Percentage of total unit of analysis*
Public	Public green space	Percentage of total unit of analysis
Institutional	Institutional green space	Percentage of total unit of analysis
Informal Urban Greenery	Informal urban green land-cover such as street trees and other greenery, roadside verges, ruderal vegetation.	Percentage of total unit of analysis
Peri-urban	Land-use outside of urban and suburban areas.	Percentage of total unit of analysis
Domestic green cover	Domestic green-space that is vegetation or water	Percentage of total unit of analysis
Domestic built cover	Domestic green-space that is built surface cover	Percentage of total unit of analysis
Public green cover	Public green-space that is vegetation or water	Percentage of total unit of analysis
Public built cover	Public green-space that is built surface cover	Percentage of total unit of analysis
Institutional green cover	Institutional green-space that is vegetation or water	Percentage of total unit of analysis
Institutional built cover	Institutional green-space that is built surface cover	Percentage of total unit of analysis
Peri-urban green cover	Peri-urban land-use that is vegetation or water	Percentage of total unit of analysis
Peri-urban built cover	Peri-urban land-use that is built surface cover	Percentage of total unit of analysis
Domestic MPA	Mean patch area of domestic green-space	m^2^
Public MPA	Mean patch area of public green-space	m^2^
Institutional MPA	Mean patch area of institutional green-space	m^2^
Peri-urban MPA	Mean patch area of peri-urban green-space	m^2^
Informal Urban Greenery MPA	Mean patch area of informal urban greenery	m^2^
Buildings cover	Proportion of land-cover by buildings	Percentage of total unit of analysis
Buildings density	Number of buildings	Count for the unit of analysis
Major road density	Distance of all major roads within the unit of analysis	m 1000 m^-2^
Minor road density	Distance of all minor roads within the unit of analysis	m 1000 m^⁻2^

*0.5, 1 or 2 km^2^ zones

In addition to the above, for models in which vegetation type was deemed to be of particular relevance (i.e. where mean LST, nitrogen dioxide and vNDVI were the dependent variables), combinations of all land-use and land-cover classes (proportion of the unit of analysis that is e.g. tree canopy in public parks or ground layer vegetation in the urban fabric) were entered as independent variables. For analyses with mean nitrogen dioxide as the dependent variable, density (m 1000 m⁻^2^) of major and minor roads (downloaded from OS Open Roads, 2018, [62]), were also considered as important predictors, as primary emission sources. Regression models were carried out at the 1 km^2^ level as this provided a more robust number of cases than doing so at the 2 km^2^ level whereas an unsatisfactorily high number of missing values for the variables given in Table 2 were produced when calculated at the 0.5 km^2^ level. All statistical tests were carried out in SPSS.23.

## Results

**Fig 4:**
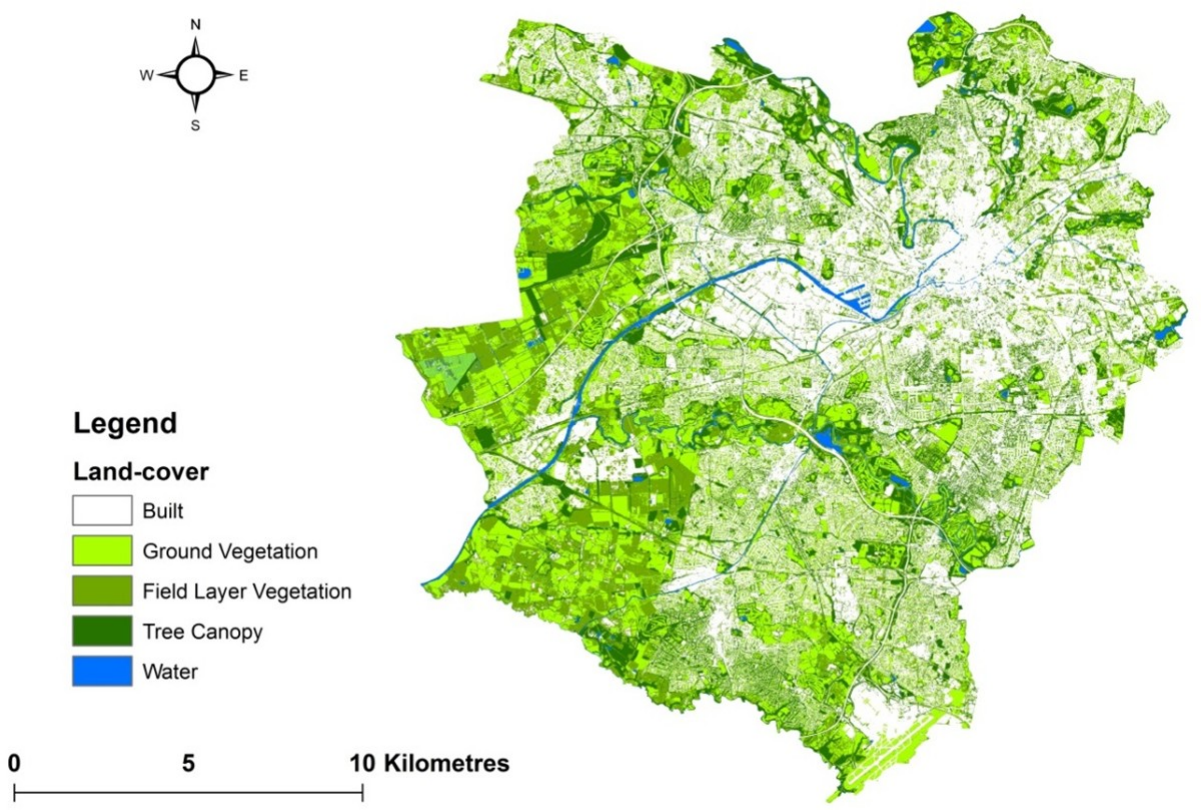
Study area characterised by land-cover (contains Planet 2017, City of Trees 2011 and OS data Crown copyright and database right 2019 Ordnance Survey (100025252)).

**Fig 5:**
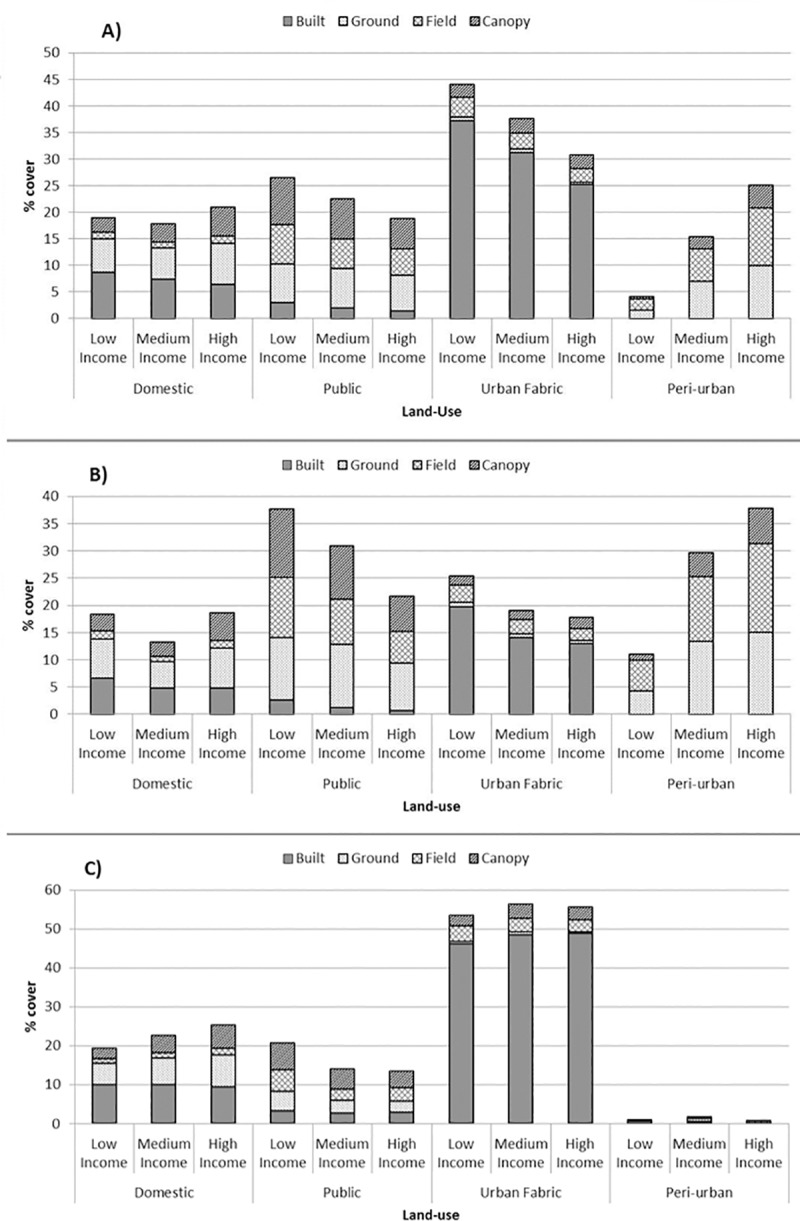
Vegetation cover within major land-uses (those comprising > 1% of the study area) A) all areas; B) low-urban areas; C) high-urban areas.

Land-cover for the study area is presented in Fig 4. The land-cover classification achieved a high level of overall accuracy (92%; Cohen’s Kappa = 0.89, p < 0.001). Fig 5 gives the relative cover by major land-uses (those comprising > 1% of the study area) and associated land-cover across low-, medium- and high-income levels (for whole-landscape and for low versus high-urban areas) at the 0.5 km^2^ level.

The spatial extent and content of public and domestic green-spaces exhibited contrasting mean values between low- and high-urban areas. Values associated with domestic gardens showed considerable variation as a function of income. For example, in terms of domestic green-space, low-urban areas contained lower overall cover relative to high-urban areas and, within the context of the latter, higher income was associated with both a larger spatial extent and a greater proportion of green land-cover. For both levels of urbanity, lower-income areas contained the greatest public green-space cover with a higher degree of surface sealing seen for this land-use in the high-urban context. Table 3 gives correlation co-efficients (Pearson’s r) between land-use types and key indicators of urbanisation.

**Table 3:** Correlations between land-use and urban indicators (at 1 km^2^).

	Low-urban					High-urban				
Green-space type	Minor Rd Density	Major Rd Density	Population Density	Buildings Density	Mean Building Size	Minor Rd Density	Major Rd Density	Population Density	Buildings Density	Mean Building Size
Domestic	0.886**	-0.042	0.802**	0.932**	-0.228**	0.552**	-0.376**	0.546**	0.955**	-0.694**
Public	0.023	0.140	0.053	0.014	0.016	-0.493**	-0.126	-0.455**	-0.401**	-0.114
Institutional	0.504**	0.217*	0.590**	0.504**	-0.055	0.247**	-0.026	0.260**	0.152	-0.192*
Urban Fabric	0.740**	0.359**	0.727**	0.713**	0.082	-0.168	0.435**	-0.214*	-0.619**	0.738**
Peri-urban	-0.725**	-0.213*	-0.710**	-0.726**	0.064	-0.311**	0.108	-0.252**	-0.237**	0.268**

* significant at the p < 0.05 level

** significant at the p < 0.01 level

**Fig 6:**
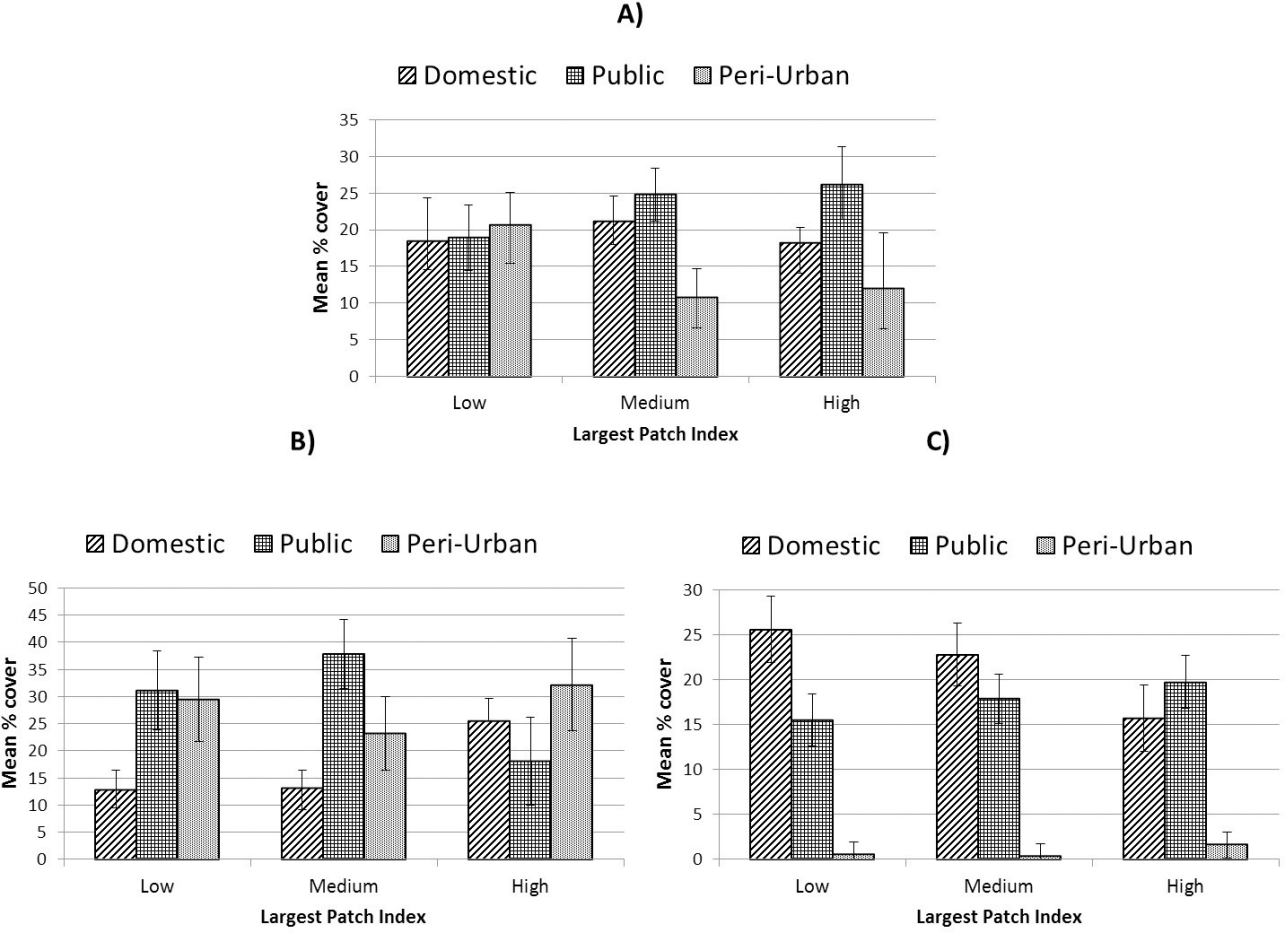
Relative extent of public, domestic and peri-urban green-space at units of 1 km^2^ across a gradient of land sharing-sparing for A) all areas; B) low-urban areas and C) high urban areas. Error bars represent 95% confidence intervals.

The relative cover by major land-use types for three quantile groups of the Largest Patch Index metric within 1 km^2^ zones (low LPI = land-sharing; high LPI = land-sparing), controlling for overall green land-cover, is presented in Fig 6. Cover by public and private land-use in land-sharing and land-sparing areas varied as a function of urbanity with public green-space contributing to land-sparing in high-urban areas but exhibiting the inverse association in low-urban areas.

**Fig 7:**
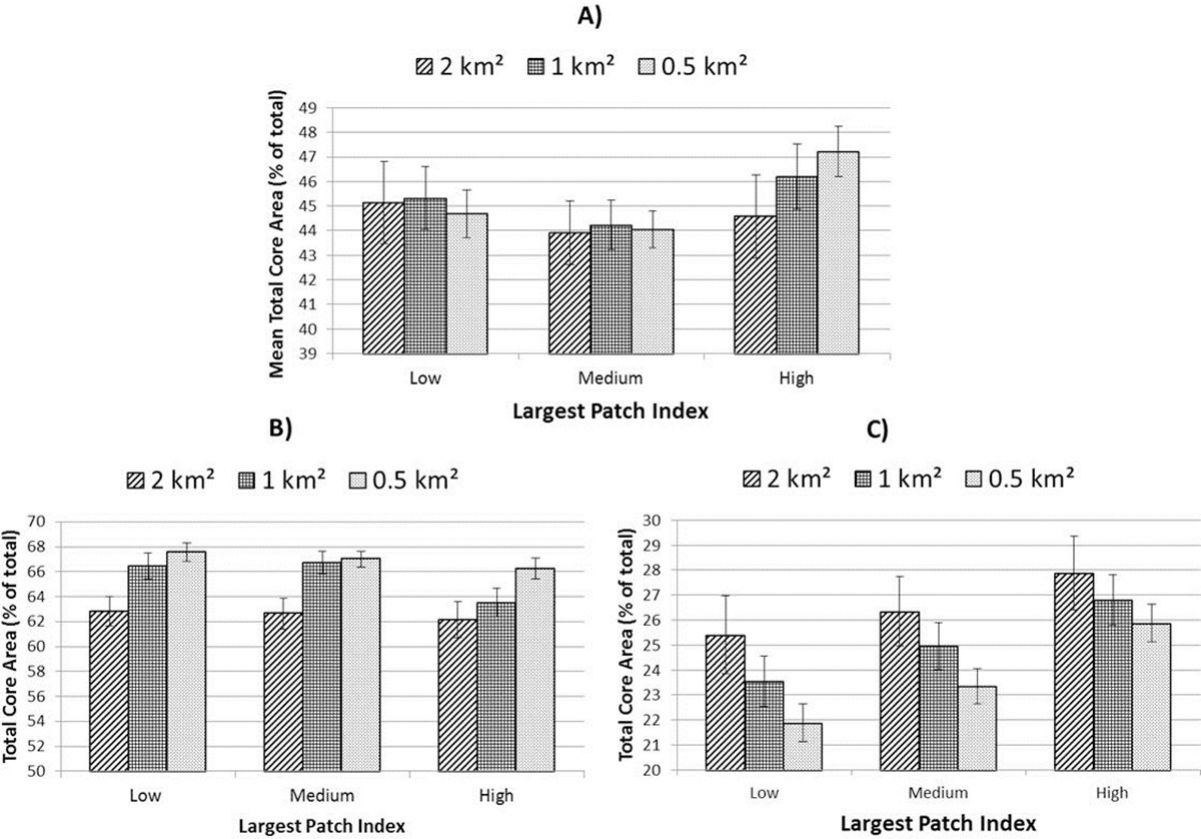
Mean Total Core Area for three levels of land-sharing-sparing controlling for overall green cover. A) all areas; B) low-urban areas and C) high urban areas. Error bars represent 95% confidence intervals.

Ecological and socio-environmental characteristics varied significantly as a function of land-sharing-sparing and urbanity. TCA, vNDVI and LST exhibited similar patterns with all three metrics displaying contrasting results between low- and high-urban areas. For TCA and LST this effect was particularly pronounced such that, for the whole-landscape assessment, areas defined as neither land-sharing nor land-sparing appeared to provide the most (LST) or least (TCA) desirable conditions, though this was not the case when assessed at either level of urbanity. Results for TCA are given in Fig 7 (see S1 and S2 Figs for LST and vNDVI results).

**Fig 8:**
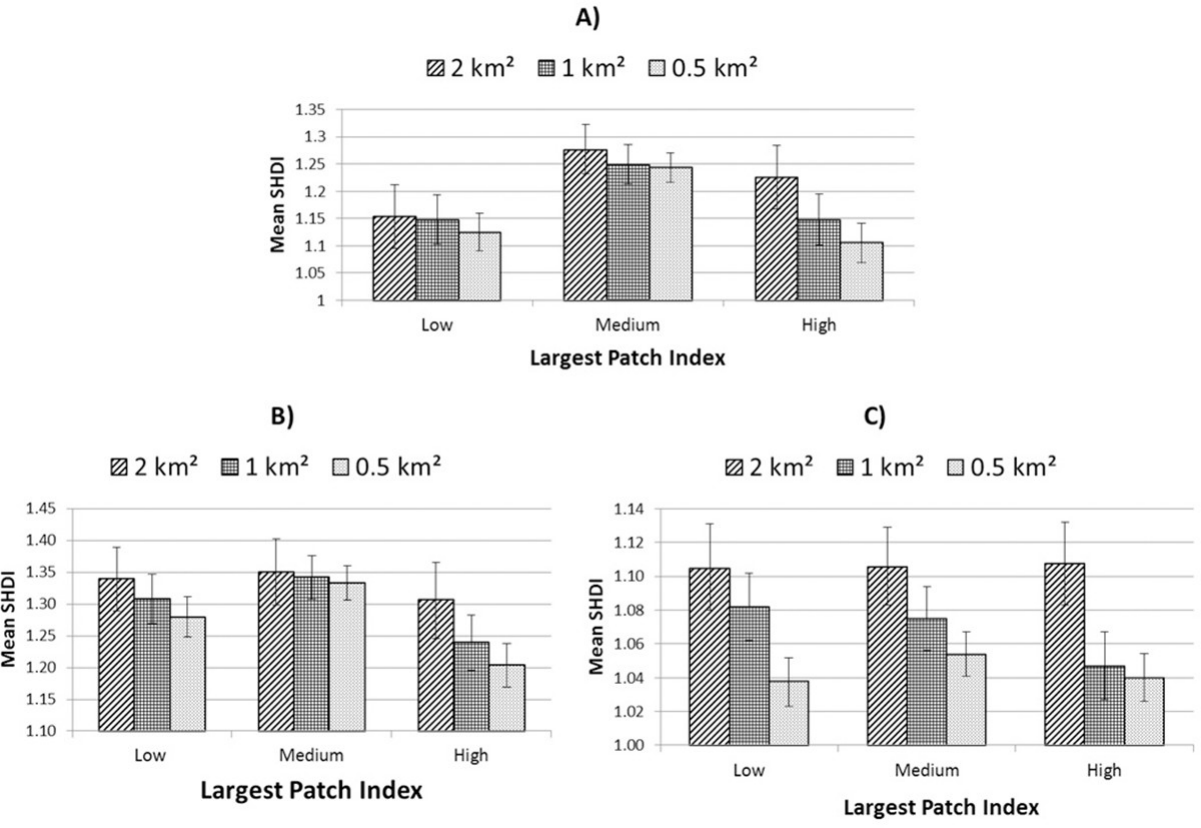
Mean SHDI for three levels of land-sharing-sparing controlling for overall green cover. A) all areas; B) low-urban areas and C) high-urban areas. Error bars represent 95% confidence intervals.

**Fig 9:**
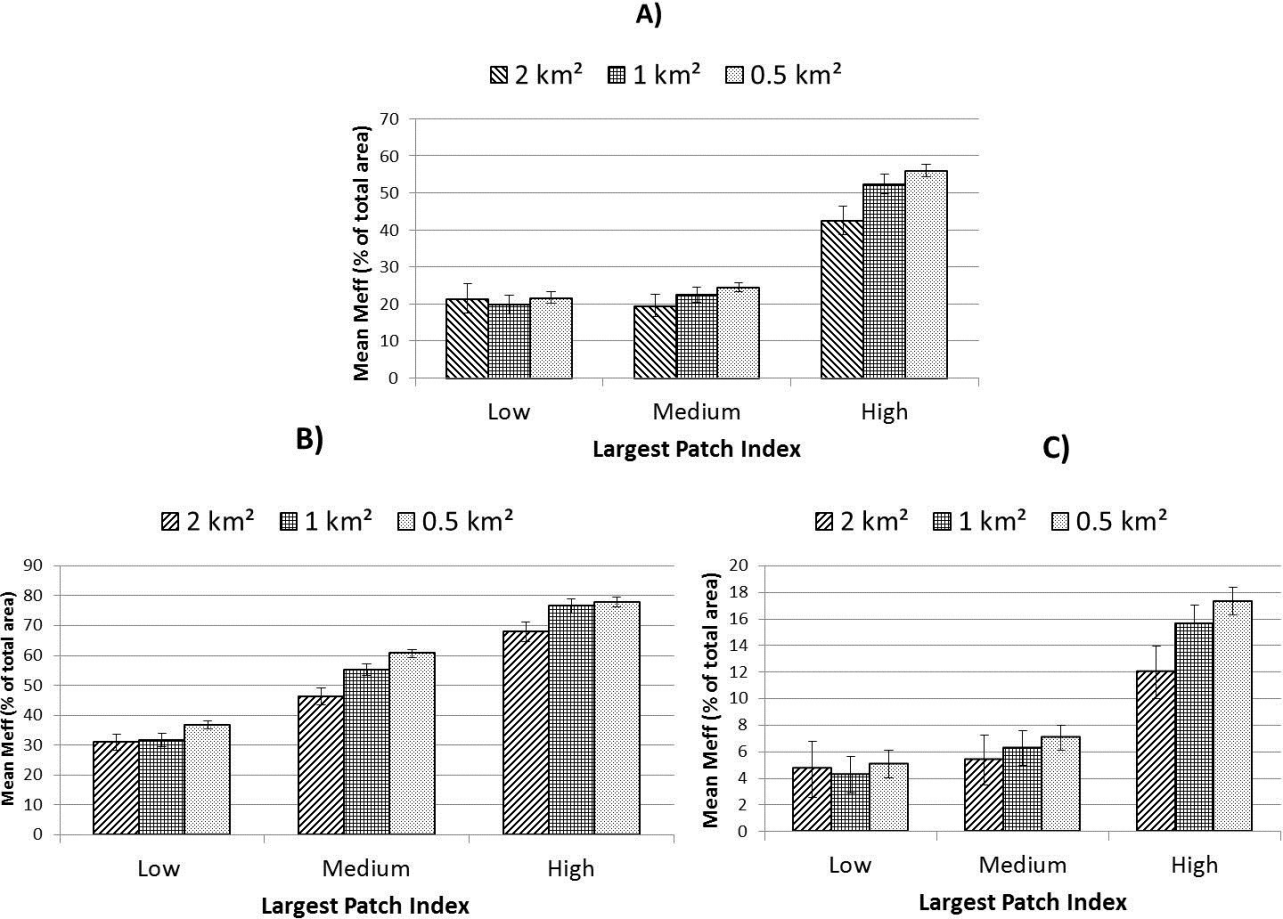
Effective mesh size for three levels of land-sharing-sparing controlling for overall green cover. A) all areas; B) low-urban areas and C) high-urban areas. Error bars represent 95% confidence intervals.

SHDI was unique in exhibiting most desirable results within areas characterised by neither land-sparing nor land-sharing for both levels of urbanity considered (Fig 8). Meff revealed consistent relationships with land-sharing-sparing regardless of context. For all three contexts (low urban, high urban and whole landscape) Meff was maximised with increased land-sparing scenarios (Fig 9). In contrast, population proximity to green-space was lowest in land-sparing scenarios at high levels of urbanity (S3 Fig), although statistical significance was not observed in low-urban areas (Table 4).

**Table 4:** Significance levels (*p* values) for all general linear model analyses carried out in this study.

	All areas			Low-urban			High-urban		
Dependent variable*	0.5 km^2^	1 km^2^	2 km^2^	0.5 km^2^	1 km^2^	2 km^2^	0.5 km^2^	1 km^2^	2 km^2^
TCA	< 0.001	0.049	0.459	0.144	<0.001	0.801	< 0.001	< 0.001	0.100
Meff	< 0.001	< 0.001	< 0.001	< 0.001	< 0.001	<0.001	< 0.001	< 0.001	< 0.001
SHDI	< 0.001	< 0.001	0.003	< 0.001	0.003	0.617	0.163	0.050	0.991
Mean temperature	0.005	0.160	0.234	0.020	0.002	0.040	0.003	0.108	0.025
vNDVI	< 0.001	<0.001	0.002	0.006	0.002	0.228	< 0.001	0.072	0.301
Nitrogen dioxide	0.004	0.070	0.045	< 0.001	< 0.001	0.007	0.033	0.187	0.936
Population <300 m to green space	0.005	0.073	0.083	0.629	0.496	0.977	0.05	0.005	0.002

*TCA: total core area; Meff: effective mesh size; SHDI: Shannon’s diversity index; vNDVI: mean normalised vegetation index of vegetation-classified pixels.

**Fig 10:**
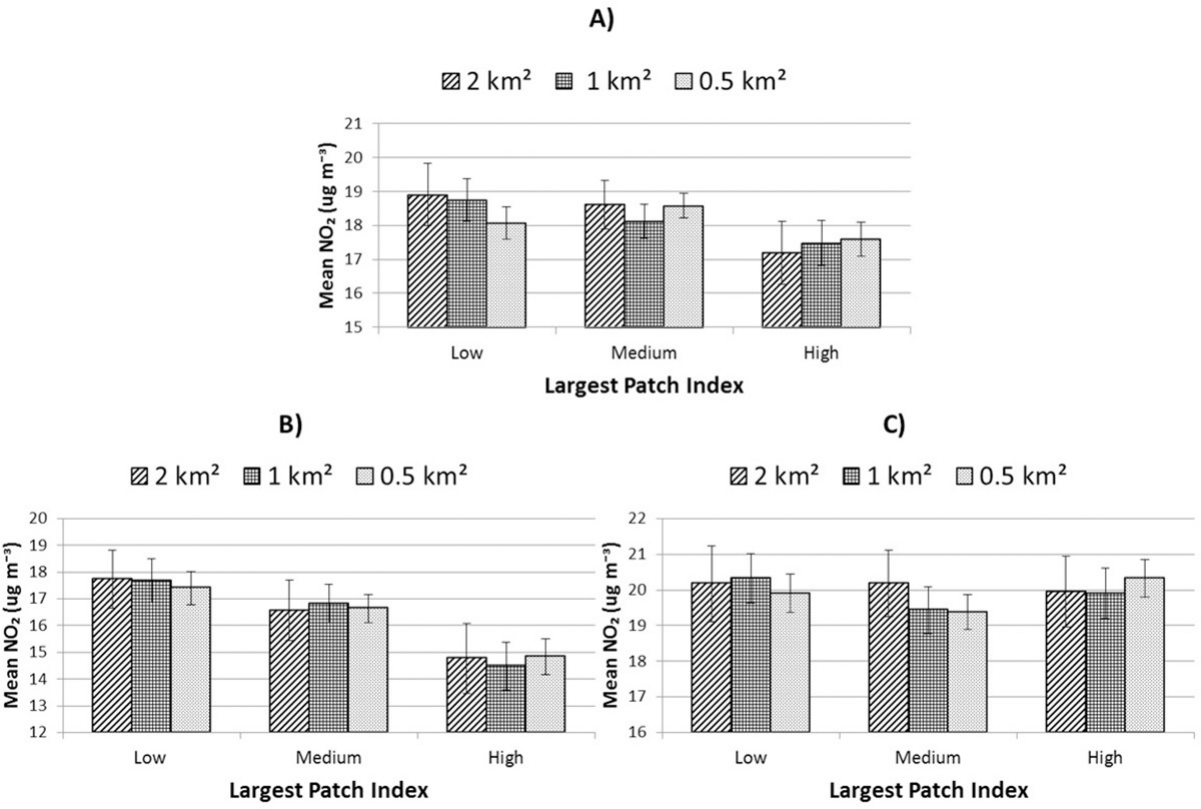
Mean ambient nitrogen dioxide concentration for three levels of land-sharing-sparing controlling for overall green cover. A) all areas; B) low-urban areas and C) high-urban areas. Error bars represent 95% confidence intervals.

Mean nitrogen dioxide concentrations for land-sharing versus land-sparing scenarios showed considerable variation as a function of both urbanity and scale. However in high-urban areas, those subject to highest overall concentrations, only the model at 0.5 km^2^ was significant (Table 4) for which land-sparing scenarios exhibited highest values (Fig 10).

Table 4 gives significance levels for models at each scale and level of urbanity considered. Overall, analyses at units of 0.5 km^2^ provided the greatest number of statistically significant tests, though low-urban areas did not follow this trend as closely as high-urban areas.

### Multiple linear regression results

Table 5 gives the results of the multiple linear regression models with landscape metrics LPI, TCA, Meff, SHDI and vNDVI as dependent variables and Table 6 summarizes regression results where socio-environmental variables mean LST, mean nitrogen dioxide concentration and total population within 300 m of a recreational green space are the dependent variables.

**Table 5:** Results of regressing land-use-land-cover attributes on landscape metrics used in this study. All tests carried out at 1 km^2^ units.

Low-urban	Beta	Sig.	High-urban	Beta	Sig.
**LPI 1 km**^**2**^					
r^2^: 0.64			r^2^: 0.47		
Major road density	-0.510	< 0.01	Major road density	-0.228	0.002
Domestic green cover	0.321	< 0.01	Domestic green cover	0.707	<0.01
Domestic built cover	-0.808	< 0.01	Domestic built cover	-0.689	< 0.01
Public built cover	-0.114	0.036	Public green cover	0.360	< 0.01
			Peri-urban green cover	0.180	0.008
**TCA 1 km**^**2**^					
r^2^: 0.89			r^2^: 0.98		
Major road density	-0.169	< 0.01	Domestic built cover	-0.080	< 0.01
Domestic built cover	-0.874	< 0.01	Public green cover	0.808	< 0.01
Public built cover	-0.284	< 0.01	Peri-urban green cover	0.451	< 0.01
Peri-urban mean patch area	0.96	0.002	Public mean patch area	0.058	< 0.01
Public green cover	0.060	0.041	Institutional green cover	0.177	< 0.01
			Domestic green cover	0.596	< 0.01
			Informal urban greenery	0.210	< 0.01
**Meff 1 km**^**2**^					
r^2^: 0.82			r^2^: 0.67		
Domestic built cover	-0.808	< 0.01	Domestic built cover	-0.664	< 0.01
Major rd density	-0.458	< 0.01	Public green cover	0.514	< 0.01
Domestic MPA	0.160	< 0.01	Peri-urban green	0.282	< 0.01
Public built cover	-0.224	< 0.01	Domestic green cover	0.942	< 0.01
**SHDI 1 km**^**2**^					
r^2^ = 0.55			r^2^ = 0.92		
Peri-urban	-0.756	< 0.01	Informal Urban Greenery	0.257	< 0.01
Informal Urban Greenery	0.237	0.01	Public green cover	0.793	< 0.01
Domestic	-0.290	< 0.01	Domestic green cover	0.712	<0.01
			Public mean patch area	-0.067	0.029
			Peri-urban	0.334	< 0.01
			Institutional green cover	0.210	<0.01
**vNDVI 1km**^**2**^					
r^2^: 0.64			r^2^: 0.75		
Public	0.393	< 0.01	Domestic field	0.251	< 0.01
Domestic built cover	-0.281	< 0.01	Domestic canopy	0.360	< 0.01
Public built	-0.134	0.024	Public field	0.252	< 0.01
Public canopy	0.241	< 0.01	Public canopy	0.399	< 0.01
Peri-urban canopy	0.513	< 0.01	Institutional field layer	0.112	0.018
Domestic mean patch area	0.167	< 0.01	Public built cover	-0.137	0.013
Public mean patch area	0.141	0.013	Major road density	-0.112	0.027
Peri-urban mean patch area	-0.367	< 0.01	Public mean patch area	0.166	< 0.01
			Public ground	0.226	< 0.01

**Table 6:** Results of regressing land-use-land-cover attributes on socio-environmental metrics used in this study. All tests carried out at 1 km^2^ units.

Low-urban	Beta	Sig.	High-urban	Beta	Sig.
**Mean LST**					
r^2^ = 0.68			r^2^ = 0.67		
Public ground	0.311	< 0.01	Urban water	-0.324	< 0.01
Urban water	-0.182	< 0.01	Major road density	-0.215	< 0.01
Minor road density	0.375	< 0.01	Public canopy	-0.338	< 0.01
Public canopy	-0.425	< 0.01	Informal Urban Greenery mean patch area	-0.405	<0.01
Peri-urban canopy	-0.632	< 0.01	Public field layer vegetation	-0.264	< 0.01
Informal Urban Greenery	-0.162	0.19	Domestic canopy	-0.529	< 0.01
Peri-urban mean patch area	-0.160	0.013	Institutional canopy	-0.206	0.027
Peri-urban mean patch area	0.187	< 0.01	Domestic mean patch area	-0.295	< 0.01
Public mean patch area	-0.125	0.022	Public water	-0.109	< 0.01
Domestic canopy	-0.210	< 0.01			
Public field layer vegetation	-0.265	< 0.01			
**Nitrogen dioxide**					
r^2^ = 0.59			r^2^ = 0.66		
Major road density	0.259	< 0.01	Major road density	0.382	< 0.01
Peri-urban field layer	-0.496	< 0.01	Peri-urban mean patch area	-0.184	< 0.01
Public canopy	0.274	< 0.01	Institutional built	0.234	< 0.01
Domestic mean patch area	-0.200	< 0.01	Domestic green cover	-0.465	< 0.01
Public field layer	-0.208	< 0.01	Institutional field layer	-0.234	< 0.01
Buildings density	0.147	0.016	Informal Urban Greenery	0.223	< 0.01
			Minor road density	0.332	< 0.01
**Pop < 300 m green-space**					
r^2^ = 0.78			r^2^ = 0.59		
Domestic built cover	0.791	< 0.01	Domestic built cover	0.390	< 0.01
Institutional built cover	0.249		Minor road density	0.483	< 0.01
Domestic mean patch area	-0.187	0.09	Domestic mean patch area	-0.295	0.018
			Public mean patch area	0.162	< 0.01

Regression analyses demonstrated that public and private land-uses exhibited unique and contrasting associations with ecological and socio-environmental variables implying considerable potential trade-offs. Moreover, these associations varied as a function of the level of urbanity and appeared to be modified by patch characteristics (mean area and green land-cover).

## Discussion

### Land-use characteristics and sharing-sparing scenarios

For the study area as a whole, and in areas of high urbanity, the distribution of public versus private green-spaces, controlling for total green land-cover, exhibited patterns that fulfill expectations of land-sharing and sparing scenarios. Mean cover of public relative to domestic green-space increased with increasing LPI (Fig 5A and 5C). However, in areas of low urbanity this pattern was not replicated where a dominance of public over domestic land-use was seen in land-sharing areas (i.e. low LPI) with domestic green-space cover highest in land-sparing areas. Our analysis suggests, therefore, that the definition of land-sharing and sparing within an urban planning context, in terms of primary land-uses which support this dichotomy, is subject to some fluidity as a function of urbanity. Moreover, the regression results highlighted domestic green and built land-covers as critical factors contributing to the largest patch index in both low- and high-urban areas, seemingly exerting a stronger influence on LPI than public green-space (Table 5). This is an important observation as it challenges some of the assumptions surrounding the patterns that result from the prevalence of public or private green spaces within green infrastructure planning frameworks [6]. That the ratio of built-to-green land-cover in domestic green-space was also shaped by socio-economic status (Fig 5) suggests that overall urbanity, land-cover and economic status may all comprise determinants of land-sharing-sparing configurations in city regions.

### Influence of land-cover

Regression analyses of individual land-use and land-cover attributes on environmental and ecological variables demonstrated a high degree of consistency between areas of contrasting urbanity though exceptions, related to SHDI in particular, were observed (Table 5). Specifically, both peri-urban and domestic land-use exhibited contrasting directions of association with SHDI dependent on whether they were assessed at low or high-urbanity. The cover by, and level of vegetation within, domestic gardens in particular were also subject to stark contrasts between areas of low- and high-urbanity (Fig 5). These disparities appeared to be underpinned by socio-economic processes. The latter, therefore, proved also to be an important local consideration moderating the influence, of land-use-land-cover combinations on ecological and environmental variables.

Cover by gardens and land-cover within gardens exhibited strong links with all socio-environmental characteristics measured. Of all land-cover types, mean LST was most strongly (negatively) associated with canopy cover in gardens in high-urban areas (Table 6), suggesting that management of domestic greening presents opportunities for climate resilience in cities. Green land-cover within informal and other private (institutional) settings also exerted significant influence on both ecological and environmental characteristics, particularly in high urban areas. This underlines the complex mosaic of land-uses contributing to effective urban green infrastructure and the need for land management within such spaces to be acknowledged as key components of planning for sustainable and resilient cities. Gardens also appeared to exert an influence on proximity to green-space and air quality. For example, domestic garden built cover was more positively associated with access to green-space in high-urban areas than was public green-space (to which category green recreational spaces belonged). This, along with the strong association observed between green-space access and minor road density, suggests that, for the current study area at least, access to recreational green spaces may be more closely related to population distribution and urban form than to provision of green-space *per se*. This is supported by the fact that domestic green-space mean patch size–denoting lower housing (and, therefore, population) density–was negatively associated with proximity to recreational green-space at both levels of urbanity (Table 6). This pattern supports other work on urban land-sparing which highlights the merits of land-sharing configurations on green-space use [34]. It also suggests, however, that increasing urban residential density, through compaction and in-filling may offer opportunities for sparing non-developed land whilst ensuring local access to green-space.

In terms of air quality, domestic garden cover showed a surprising negative association with mean nitrogen dioxide concentrations: the strongest of all land-uses types for high urban areas. Specific land-covers within gardens did not seem to be responsible for this association (Table 6), but that garden cover correlated negatively (p < 0.01) with density of major roads (Table 3) may offer a potential explanation and suggests urban form, rather than land-cover, as a critical factor. This idea is supported by results reported elsewhere which suggest that complex geometric patterns created by fragmented urban forms may reduce traffic-related congestion and pollution [63]. That tree cover in public green spaces in low-urban areas was positively associated with mean nitrogen dioxide concentrations may explain to some degree why public green-space cover overall was not statistically relevant to mean nitrogen dioxide concentrations. This stands in contrast to findings in other studies highlighting the ability of trees to remove nitrogen dioxide from the environment [43]. However, ours is the first study of its kind to consider a range of vegetation types across different land-uses simultaneously. The results of our regression models showed that tree canopy and lower vegetation types exhibited contrasting associations with levels of nitrogen dioxide, with field layer vegetation showing the greatest negative influence on ambient nitrogen dioxide at both levels of urbanity. Broader evidence on the relationship between the urban canopy and ambient nitrogen dioxide is, however, mixed [64] and known to be subject to meteorological factors [65]. Specifically, ambient nitrogen dioxide has been shown to decrease with increasing local air temperatures (Ibid.). The latter is particularly relevant given that tree cover was negatively associated with LST in our results and implies a potential trade-off resulting from different socio-environmental outcomes related to the presence of green infrastructure (i.e. urban cooling and air quality). Overall, cover by water in urban areas suggested the greatest cooling effect by any land-cover, underlining the importance of waterways and wetlands in the regulation of the urban micro-climate (e.g. [66]).

### Level of urbanity

Our analysis suggests that complex trade-offs may be implied by the ascendency of one or other of a land-sharing versus land-sparing approach within different contexts of urbanisation. This appeared to be most evident for socio-environmental factors considered. For example, models for mean LST and nitrogen dioxide values exhibited differing trends between high and low areas of urbanity. This mirrored similarly inverse trends related to domestic green-space cover, presenting the latter as a potential causal factor. In terms of access to green-space land-sharing-sparing configurations only appeared to be relevant in high-urban areas. Vegetation quality (vNDVI) exhibited highest mean values within land-sharing scenarios in low-urban areas (0.5 and 1 km^2^, S2 Fig) whereas, in high-urban areas, highest values were associated with land-sparing.

Although the two levels of urbanity presented some contrasting results, there was evidence of some consistency related to specific spatial or class-level components. For example, regardless of scale or level of urbanity, land-sparing appeared consistently to promote greater connectivity (Meff). That Meff was highest in land-sparing scenarios in both urbanity contexts (even though this implied different land-use patterns) suggests that land-use is a minor consideration relative to land-cover and spatial characteristics when aiming at connectivity. In the case of total population in close proximity to a recreational green space, analysis of high-urban areas suggested provision was consistently lowest in land-sparing environments at all scales. This contrast between outcomes for Meff and proximity to green-space suggests a potential trade-off between meeting separate ecological and societal goals through land-sharing-sparing approaches in the most urbanised areas. In terms of land-cover, tree canopy consistently promoted greater cooling (lower mean LST) and greater vegetation vigour, regardless of land-use or urbanity. This implies that, as identified by others [9], restoration through afforestation may effectively support broader landscape considerations in the promotion of urban ecosystem services and their resilience. From the perspective of landscape heterogeneity, differences in SHDI were significant between sharing and sparing scenarios in low-urban areas at the 0.5 and 1 km^2^ scale. At these scales, areas which comprised neither sharing nor sparing configurations exhibited greatest land-cover diversity, with land-sharing areas also showing significantly greater mean SHDI values than land-sparing areas (Fig 8). In addition, in low-urban areas peri-urban land-use appeared to play a detrimental role in landscape heterogeneity (Table 5). Overall, therefore, our results point towards an increase in vegetation diversity and quality in areas characterised by peri-urban land-use through the introduction of more typically urban green-space types (Figs 5, 6 and 9). In the high-urban context, all major green land-uses appeared to contribute to landscape heterogeneity (Table 5) suggesting that increases in green land-cover of any type are beneficial regardless of land-sharing-sparing considerations (which were not statistically relevant to SHDI in high urban areas, Table 4).

### Scale

Associations between ecological and socio-environmental patterns and land-sharing-sparing scenarios appeared to be moderated as a function of the scale of investigation employed. For example, for the study area as a whole, when measured at units of 2 km^2^, TCA appeared to be highest within spatial configurations which represent land-sparing scenarios (Fig 6). In contrast, land-sparing appeared to promote this critical landscape characteristic when measured at scales of ≤ 1 km^2^. The influence of scale differed between variables. For example, of the landscape attributes tested, SHDI exhibited generally higher values when measured at larger scales, whereas (standardised) Meff values were highest at smaller scales of investigation. In terms of levels of statistical relevance, our analysis exhibited scale-dependence (Table 4). This is important from both an urban planning and nature conservation perspective. When treating the study area landscape as a whole, a greater incidence of statistical significance was exhibited at smaller scales of investigation for most variables considered (Table 4), though urbanity appeared to mediate this trend. For example, in low-urban areas, analyses at a scale of 1 km^2^ returned the greatest number of statistically significant tests, whereas in high-urban areas this occurred at the 0.5 km^2^ scale. This implies that in more highly fragmented landscapes, higher spatial resolution is necessary to discern land-sharing-sparing associations with environmental characteristics.

This variance as a function of scale and urbanity poses a challenge for landscape analysis which would inform decisions on social and ecological goals respectively. For example, analyses of species distributions in urban ecological studies are commonly carried out at units of 1 x 1 km^2^ [53], [54] though our results suggest that working at such scales may not capture the potential for land-cover configurations to similarly achieve co-benefits such as urban cooling. Therefore, using a multi-scale approach such as that developed here, considering multiple socio-environmental characteristics relevant to sustainable urban development may be of considerable merit. This is largely due to the possibility, as demonstrated here, of identifying optimum scales of analysis through relatively rapid assessments using GIS and remote sensing techniques.

### Moving the land-sharing-sparing debate forward in urban areas

The analysis presented here demonstrates how a landscape approach, incorporating spatially coincident measures of land-use and land-cover, can be employed to unpick spatial and ecological complexities relevant to sustainable urban development. Our analysis suggests three pathways for future evaluation and research on landscapes subject to the process of urbanization. Firstly, scale (spatial units) should be considered in planning and research where multiple socio-environmental concerns are to be addressed. In the case of the former, we suggest that a modular approach working at smaller, local scales of analysis should be employed to capture variables that are highly spatially sensitive. Concurrently, research should focus on evaluating the potential for up-scaling analyses of small-scale phenomena (e.g. micro-climate regulation) to align with larger theoretically established units of investigation of others (e.g. species distribution). Secondly, spatial context in terms of level of urbanity should be equally considered as a significant mediating factor in the determination of optimal land-use configurations. Not only do levels of urbanization modify the spatial characteristics of landscapes, but from the perspective of landscape resilience and ecosystem services provision, different contexts will dictate the nature of management goals related to spatial planning. For example, in urban areas where natural green cover is highly fragmented but may also exhibit high heterogeneity, developing landscape configurations which increase connectivity per unit area may take priority over increasing diversity. Conversely, in peri-urban areas where green cover consists of larger and more connected, but highly homogenous (e.g. due to agricultural practices) patches, land-use-land-cover combinations which promote landscape complexity rather than cohesion may be prioritised. Further, our results suggest that even when different landscape configurations are promoted in urban and peri-urban areas, this may in reality involve the parallel promotion of similar land-use types. However, we concede that the current study used a highly simplified dichotomous take on an urban-to-peri-urban gradient, controlling for overall green land-cover within each zone. In reality urban-rural gradients will consist of multiple degrees of urbanisation and human density. Furthermore, overall greenness of the environment and the merits of land-sharing versus sparing outcomes are likely to be subject to non-linear functional relationships [24]. Therefore, our findings should be tested, ideally across landscapes which exhibit multiple combinations of green land-cover and population, in order to identify potential thresholds in the relative performance of land-sharing-sparing configurations.

Land-use-land-cover combinations exerted a significant influence on the social-ecological-environmental characteristics explored here and exhibited the potential to subvert assumptions related to land-sharing-sparing scenarios (e.g. the relative distribution of public and private green-space). We suggest, therefore, as a third imperative for future research on land-use configurations towards sustainable urban landscapes, that land-cover specifically (and ecological restoration more broadly) be embedded within research designs as a qualitative consideration with a view to potentially clarifying and resolving tensions related to spatial considerations. Operationalising and refining these three principles of analysis could help to clarify and harness complexity in human-dominated landscapes towards spatial configurations that promote productive, diverse and ultimately resilient urban areas.

## Supporting information

S1 Appendix Calculation of land surface temperature from Landsat 8 TIRS imagery. (DOCX)

S1 Fig Mean land surface temperature for three levels of land-sharing-sparing controlling for overall green cover. A) all areas; B) low-urban areas and C) high-urban areas. Error bars represent 95% confidence intervals.
(TIF)

S2 Fig Mean vNDVI (normalised difference vegetation index for vegetation-class pixels) for three levels of land-sharing-sparing controlling for overall green cover. A) all areas; B) low-urban areas and C) high-urban areas. Error bars represent 95% confidence intervals.
(TIF)

S3 Fig Mean total population within 300 m of a green space for three levels of land-sharing-sparing controlling for overall green cover. A) all areas; B) low-urban areas and C) high-urban areas. Error bars represent 95% confidence intervals.
(TIF)

S1 Dataset 0.5 km^2^ data. (CSV)

S2 Dataset 1 km^2^ data. (CSV)

S3 Dataset 2 km^2^ data. (CSV)
