# Supplementary material for: Evaluating urban environmental and ecological landscape characteristics as a function of land-sharing-sparing, urbanity and scale
Source: PLoS One. 2019 Jul 25;14(7):e0215796. doi: 10.1371/journal.pone.0215796 (PMC6657829; doi:10.1371/journal.pone.0215796)
Supplement: S1 Appendix — (DOCX) [file pone.0215796.s001.docx]

**Evaluating urban environmental and ecological landscape characteristics as a function of land-sharing-sparing, urbanity and scale**

Dennis, M. Scaletta, K.L., and James. P

**S1 Appendix**

**Calculation of land surface temperature from Landsat 8 TIRS imagery**

The determination of land-surface temperature from Landsat TIRS 8 imagery involved six basic steps adapted from Advan and Jovanovska (2016) and employs, at different stages bands 10 (thermal infrared), 5 (near infrared) and 4 (visible red).

Step 1 is the calculation of top-of-atmosphere (TOA) reflectance according to equation (1):

*TOA* = 𝑀𝐿 ∗ Band10 + 𝐴𝐿− 𝑂𝑖 (1)

where 𝑀𝐿 represents the band-specific multiplicative rescaling factor, 𝐴𝐿 is the band-specific additive rescaling factor, and 𝑂𝑖 is the correction for Band 10 (*ML, AL and O*𝑖 are obtained from the Landsat 8 image metadata files).

In step 2, TOA spectral radiance is converted to brightness temperature (BT) using the thermal constants for band 10 as provided in the image metadata using equation (2):

$BT=\frac{K_{2}}{\ln\left[ \left( K_{1}/TOA \right)+1 \right]}-273.15$ (2)

Where K₁ and *K₂* represent band-specific thermal conversion constants provided by the image metadata. The value 273.5 represents absolute zero and is added in order to obtain results in degrees Celsius.

Step 3 is to apply a correction for emissivity (the efficiency with which a body transfers energy from its surface into the atmosphere). This requires the normalised difference vegetation index (NDVI) to be calculated according to equation (3):

$NDVI=\frac{Near Infrared \left( Band 5 \right)-Red(Band 4)}{Near Infrared\left( Band 5 \right)+Red(Band 4)}$ (3)

In step 4 (based on Carlson and Ripley, 1997) the proportion of vegetation (Pv) is calculated from the NDVI using equation (4):

$P_{v}= \left[ \frac{NDVI-{NDVI}_{min}}{{NDVI}_{max}- {NDVI}_{min}} \right]^{2}$ (4)

where NDVI is the value per pixel and ${NDVI}_{max}$ and ${NDVI}_{min}$ are the maximum and minimum NDVI values for the study area, respectively.

Step 5 is to calculate the emissivity (ɛ) using equation (5):

$\varepsilon=0.004 P_{v}+0.986$ (5)

Where 0.004 and 0.986 are constants derived from the results of Sobrino et al. (2004) and which relate to the standard deviation of soil emissivity (0.004), and mean values for soil and vegetation emissivities (0.986) calculated from the ASTER spectral library.

Step 6, the final step, is to calculate land surface temperature (LST) in Celsius using equation (6)

$LST= \frac{BT}{\left\{ 1+[\left( \frac{\lambda BT}{\rho} \right)\ln\varepsilon] \right\}}$ (6)

where BT is at-sensor brightness temperature in °C (from Step 2), λ is the wavelength of emitted radiance (i.e. of band 10), $\varepsilon$is the emissivity calculated in Step 5 and $\rho$ represents the second radiation constant:

$\rho=h\frac{c}{\sigma}=1.438 \times{10}^{-2} m K$

where $\sigma$is the Boltzmann constant (1.38 $\times{10}^{-23} J/K$), $h$ is Planck’s constant (6.626 ${10}^{-34} Js)$and *c*  is the velocity of light (2.998 $\times{10}^{8} m^{-s}$).

**References**

Avdan U, Jovanovska G. Algorithm for automated mapping of land surface temperature using LANDSAT 8 satellite data. Journal of Sensors. 2016. http://dx.doi.org/10.1155/2016/1480307

Carlson TN, Ripley DA. On the relation between NDVI, fractional vegetation cover, and leaf area index. Remote sensing of Environment. 1997 Dec 1;62(3):241-52.

Sobrino JA, Jiménez-Muñoz JC, Paolini L. Land surface temperature retrieval from LANDSAT TM 5. Remote Sensing of environment. 2004 Apr 30;90(4):434-40.
