# Supplementary figures and images for: Evaluating urban environmental and ecological landscape characteristics as a function of land-sharing-sparing, urbanity and scale
Source: PLoS One. 2019 Jul 25;14(7):e0215796. doi: 10.1371/journal.pone.0215796 (PMC6657829; doi:10.1371/journal.pone.0215796)

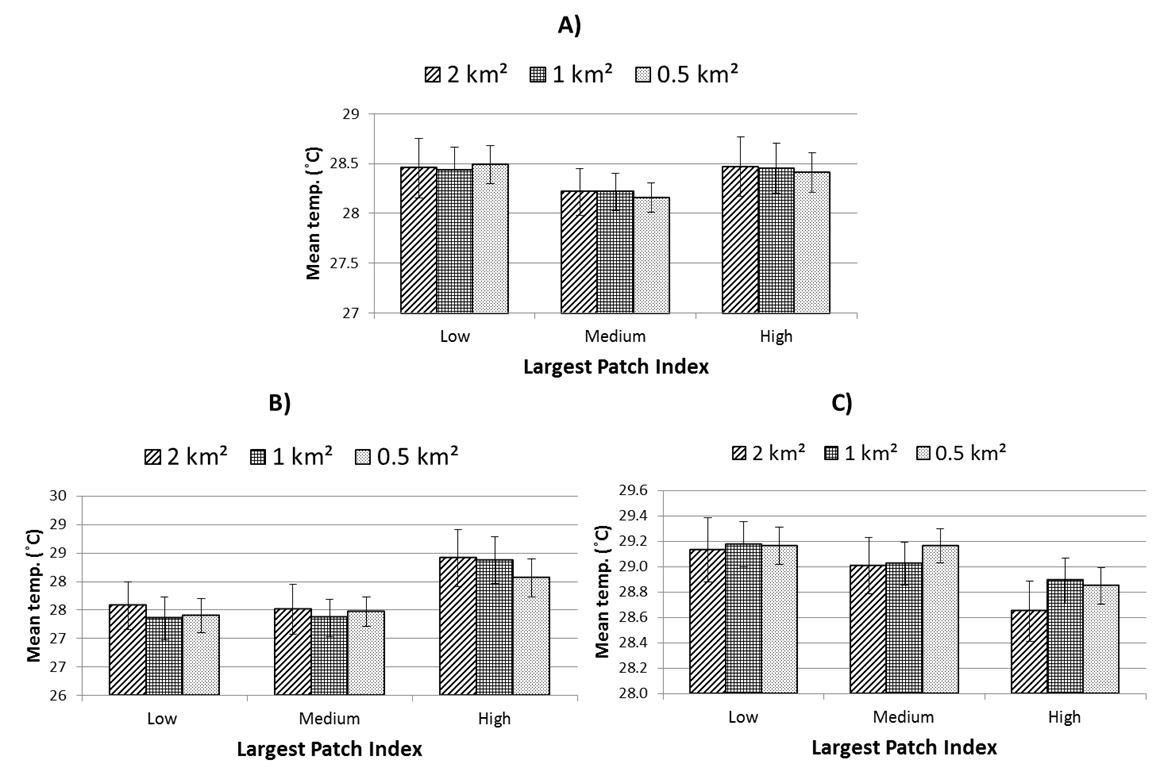

Supplement: S1 Fig — A) all areas; B) low-urban areas and C) high-urban areas. Error bars represent 95% confidence intervals. (TIF) [file pone.0215796.s002.tif]

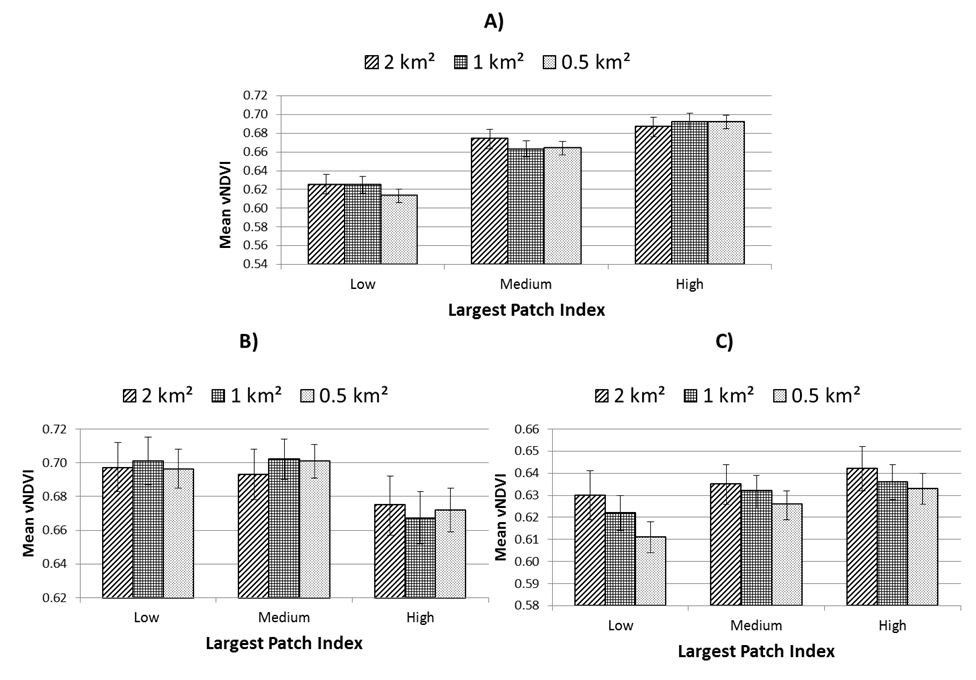

Supplement: S2 Fig — A) all areas; B) low-urban areas and C) high-urban areas. Error bars represent 95% confidence intervals. (TIF) [file pone.0215796.s003.tif]

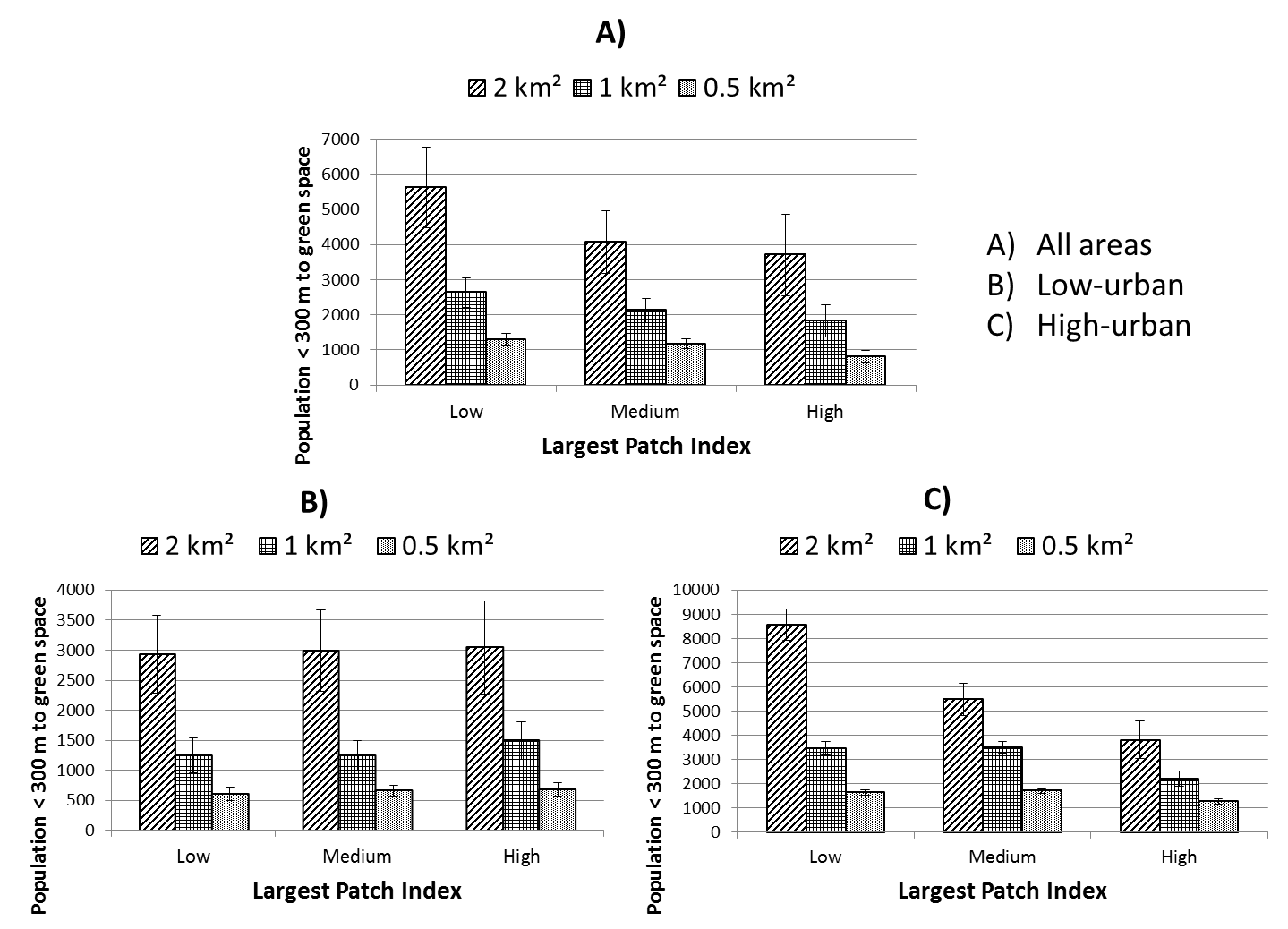

Supplement: S3 Fig — A) all areas; B) low-urban areas and C) high-urban areas. Error bars represent 95% confidence intervals. (TIF) [file pone.0215796.s004.tif]
